# Ultra-low level HIV p24 drives immune activation in antiretroviral therapy-treated people living with HIV

**DOI:** 10.1038/s43856-025-01261-3

**Published:** 2025-12-02

**Authors:** Enrico Richter, Julia König, Antonia Büning, Theresa Bechtel, Andrea Casado, Jernej Pušnik, Trevor A. Crowell, Heiko Jessen, Jürgen K. Rockstroh, Christoph Boesecke, Stefan Esser, Christoph Stephan, Hendrik Streeck

**Affiliations:** 1 Institute of Virology, University Hospital Bonn, University of Bonn, Bonn, Germany; 2 German Center for Infection Research (DZIF), partner site Bonn-Cologne, Braunschweig, Germany; 3 U.S. Military HIV Research Program, CIDR, Walter Reed Army Institute of Research, Silver Spring, MD USA; 4 Henry M. Jackson Foundation for the Advancement of Military Medicine Inc, Bethesda, MD USA; 5 Infectiology Berlin MVZ (Praxis Jessen² + Colleagues), Berlin, Germany; 6 Department of Medicine I, University Hospital Bonn, University of Bonn, Bonn, Germany; 7 HPSTD HIV Outpatient Clinic, Department of Dermatology, University Hospital Essen, University Duisburg-Essen, Essen, Germany; 8 Institute for Translational HIV Research, University Hospital Essen, University Duisburg-Essen, Essen, Germany; 9 Department of Internal Medicine 2, Infectious Diseases Unit, University Hospital Frankfurt, Goethe University Frankfurt, Frankfurt, Germany

**Keywords:** HIV infections, Chronic inflammation

## Abstract

**Background:**

Despite effective antiretroviral therapy (ART), people living with HIV (PLWH) often exhibit persistent immune activation, the mechanisms of which remain unclear. Increasing evidence suggests that residual low-level viremia and ongoing viral protein expression may persist even under long-term suppressive ART, underscoring the need for a better understanding of residual HIV persistence.

**Methods:**

We therefore optimized a digital single-molecule array (Simoa®) technology to detect ultra-low levels of HIV p24 antigen in plasma, achieving femtogram sensitivity. In addition, we used flow cytometry to analyze HIV-specific T cell responses.

**Results:**

Here we show that in a cohort of 108 participants with chronic HIV on long-term ART with HIV-1 RNA < 30 copies/mL for >4 years, p24 is detectable (17 − 370 fg/mL) in 42. Dual protease inhibitor therapy is associated with significantly lower p24 levels (p  <  0.05), while age, ART duration or CD4/CD8 ratio show no effect. Monitoring 41 individuals who initiated ART during acute HIV, p24 remains detectable in 20% after two years. Although p24 correlates with viral RNA early in ART (r = 0.83, p < 0.0001), this association is lost after two months (r = 0.20, p = 0.21). Importantly, p24+ individuals show significantly higher frequencies of PD-1 + , CD38 + , and CD38 + HLA-DR + CD8 T cells (p  <  0.01; p  <  0.05), alongside enhanced TNF-α and CD107a responses to HIV Gag (p  <  0.01; p  <  0.05).

**Conclusions:**

To best of our knowledge, our findings provide the first large-cohort evidence of low-level p24 persistence during suppressive ART and suggest that ongoing p24 production may contribute to residual immune activation in treated PLWH.

## Abstract (web summary)

Richter et al. use ultrasensitive digital ELISA to measure low-levels of the HIV capsid protein p24 in people on long-term antiretroviral therapy. They find that persistent low level p24 production is linked to increased immune activation, despite viral suppression.

## Plain language summary

HIV treatment, known as antiretroviral therapy (ART), can successfully suppress the virus in the blood, but it cannot completely remove it from the body. Many people living with HIV continue to show signs of immune system activation, even when the virus seems undetectable with current detection methods. In this study, we used an extremely sensitive test to measure a viral capsid protein called p24, a marker of ongoing HIV activity. We found that some people on long-term treatment still have very low p24 levels, and these individuals also show higher immune activation. This suggests that residual HIV activity may continue to stimulate the immune system, highlighting the need for improved treatment strategies.

## Introduction

Introducing antiretroviral therapy (ART) and its ability to effectively suppress HIV-1 replication raised hopes among researchers, clinicians and patients with enthusiasm to enable the eradication of HIV^1–3^. However, within just two years, optimism gave way to considerable pessimism. Despite the success of combination ART in suppressing viral replication and restoring immune function, ART is not curative, as most patients experience rapid viral rebound after treatment discontinuation^4^. One of the most discouraging discoveries was the persistence of latently infected cells harboring replication-competent HIV^5^. These viral reservoirs, established early during acute HIV-1 infection, remain unaffected by ART and can rapidly reignite systemic infection upon ART interruption^6^. Over time, it has become evident that these latent reservoirs represent the major barrier to HIV-1 eradication^7^.

A deeper understanding of the mechanisms sustaining infection is critical for optimizing therapy and developing curative strategies. Although ART is highly effective in suppressing viral replication, studies have shown that a small group of individuals on long-term therapy exhibit low-level, nonsuppressible viremia, typically ranging between 20 and 200 copies/mL^8,9^. Furthermore, even in individuals with HIV-1 RNA levels below the detection limit (<20–30 copies/mL), HIV transcriptional activity can persist. Using highly sensitive research assays, very low levels of plasma viremia have been detected, indicating ongoing viral transcription despite suppression^10–12^. It is still unclear whether this residual viremia results from ongoing HIV-1 replication - possibly due to poor drug penetration or immune sanctuaries - or from the gradual decay of the viral reservoir, rather than ongoing replication^13–15^. Nevertheless, persistent low-level viral replication and continuous production of HIV proteins, despite ART, underscore the need for improved treatment approaches^8,16,17^. The role of immune activation in HIV pathogenesis is highlighted by the finding that, in untreated HIV infections, T-cell activation independently predicts disease progression regardless of viral load^18–20^. Conversely, individuals with suppressed viral loads on ART have significantly lower peripheral T-cell activation compared to untreated people living with HIV (PLWH)^21–24^. However, in many cases, CD8 T cell subsets and their activation status fail to fully normalize even when viral suppression is achieved, highlighting persistent immunological perturbations in treated individuals. Indeed, incomplete reconstitution of CD4 T cell counts^25–27^, memory subset proportions, activation, and elevated exhaustion markers (CD38, HLA-DR, PD-1)^26,28–30^, as well as reduced functionality, persists even in PLWH on long-term suppressive ART^31–33^. In recent years, multiple groups have enhanced the detection of intact proviral genomes by using primers and probes targeting conserved viral regions^34–37^. However, it has been shown that the majority of viral RNA detected under ART using ultrasensitive assays is not intact and originates from defective proviruses that cannot replicate^38–41^. This raises questions about the clinical significance of low-level viremia detected in patients. In contrast, HIV proteins may readily trigger an adaptive T cell response and actively control or reduce HIV viremia^16,39^. Despite their importance, measuring HIV proteins, especially under suppressive ART, remains technically challenging due to their extremely low levels. Among these proteins, the p24 antigen is particularly significant. As HIV’s most abundant protein, p24 is essential for capsid assembly, viral replication, and clinical monitoring^42,43^. However, conventional ELISA assays lack sensitivity, with detection limits above 5 pg/mL, precluding correlation analyses at lower concentrations^44^. Over the past 15 years, advances in ultrasensitive protein detection - particularly the groundbreaking single-molecule array (Simoa®) technology - have enabled the quantification of ultra-low protein levels^45,46^. This digital ELISA offers a major advantage in measuring inducible, translationally competent virus. A recent study demonstrated that combining digital p24 readout with a quantitative viral outgrowth assay (Q-VOA) optimally tracks HIV-1 replication kinetics at ultra-low viral levels^47^.

To determine whether residual viral protein production occurs in ART-treated PLWH and drives low-level immune activation, we optimized digital ELISA with single-molecule array technology, enabling p24 detection in femtomolar concentrations. Despite successful ART for ≥4 years, we detect ultra-low p24 levels in some of the tested PLWH. p24 levels declined significantly after ART initiation but remained detectable in 20% of participants over two years. Interestingly, while dual protease inhibitor therapy leads to a significant reduction in p24 levels, switching from a three-drug regimen to a two-drug regimen does not show the same effect. Furthermore, virally suppressed PLWH with ultra-low p24 levels exhibit more activated and robust HIV-specific T-cell responses despite ongoing treatment, suggesting ongoing recognition of infected cells. Our findings indicate that persistent low-level p24 production may drive residual immune activation in ART-treated PLWH.

## Methods

### Study participants and ethics statement

Blood specimens from people without HIV were obtained from the blood donation center at University Hospital Bonn (Germany) as well as from a previous study (approved by the ethics committee board of University Hospital Bonn and the Medical Association of North Rhine-Westphalia, reference no. 216/22, 372/20, 312/22, and 2022179). Blood samples from ART-treated individuals with chronic HIV-1 (named “Chronic Cohort”) were recruited from the outpatient HIV and STD clinic at the University Hospital Essen, Germany (IRB at the University Duisburg-Essen, No. 17-7846-BO). Blood samples from people with acute and chronic HIV-1 were obtained during the RV464 study (named “RV464 Cohort” and approved by the ethics committee board of the University Hospital Bonn, reference no. 440/19). The planning, conduct, and reporting of these above-mentioned studies were performed in line with the Declaration of Helsinki, as revised in 2013. All research was conducted in accordance with relevant guidelines and regulations. All study participants provided written informed consent.

### Sample collection and storage

Three ethylenediaminetetraacetic acid (EDTA) blood collection tubes (Sarstedt) of peripheral blood (total volume of 10–25 ml) were collected from each individual by venipuncture. Additionally, different HIV uninfected specimens were obtained from the blood donation center University Hospital Bonn (Germany). Blood samples were centrifuged for 10 min at 600 × g, after which plasma was harvested and stored until analysis at −80 °C. Peripheral blood mononuclear cell (PBMC) isolation was performed via Ficoll gradient centrifugation, as previously described in ref. ^48^. After isolation, PBMCs were frozen at −80 °C overnight. For long-term storage, frozen PBMC samples were transferred to liquid nitrogen. Due to limited PBMC availability from some individuals, the sample sizes vary between experiments and panels.

### HIV-1 RNA detection and quantitation

HIV-1 RNA was detected and quantified via the Alinity mHIV-1 AMP Kit (Abbott). HIV-1 was isolated from 600 μL of plasma sample according to the manufacturer’s instructions. Samples below the established cutoff of 30 copies/ml were considered as not quantifiable viral load and classified as fully virologically suppressed in the context of standard research and clinical treatment definitions. The Alinity mHIV-1 assay primers and probes target the integrase and LTR regions of the viral genome.

### Ultrasensitive digital immunoassay for HIV‑1 p24

Plasma samples were thawed either at room temperature or overnight at 4 °C. To increase the sensitivity of p24 antigen detection, immune complex dissociation was performed prior to p24 quantification. Therefore, plasma samples were diluted 1:1 with 1.5 M glycine-HCl (pH 2.5), incubated at 37 °C for 60 min, and then neutralized to pH 7.0 using 1.5 M Tris-HCl (pH 9.0). Next, diluted plasma samples were centrifuged at 10.000 x g for 5 min, and the plasma concentration of HIV-1 Gag p24 was determined on a Simoa® SR-X analyzer using the Simoa® HIV p24 kit (Quanterix) following the manufacturer’s protocol. In brief, a total of 125 μl of each plasma sample, reference calibrators, and quality controls were loaded into a 96-well plate supplied with the Simoa® SR-X Disk Kit (Quanterix). Next, samples were mixed with detector solution as well as beads and placed the plate on an orbital shaker to incubate at 30 °C with shaking at 800 rpm for 45 min. Afterwards, samples were washed on a magnet washer, a streptavidin, β-galactosidase conjugate was added to each well, and samples were incubated again at 30 °C with shaking at 800 rpm for 10 min. After repeated washing steps, the 96-well plate was loaded into the SR-X analyzer and the HIV p24 analysis protocol was performed to determine p24 levels. Reference calibrators for the establishment of the standard curve, two quality control samples of low and high concentration as well as three known HIV negative plasma samples, were included in each of the runs to ensure consistent performance of the digital assay throughout the study. In addition, each plasma sample was measured in triplicates. Post-run quality control checks included but are not limited to confirmation of the standard curve, quality controls, sample CVs (<20%), and any relevant errors during the analysis as per manufacturer’s instructions. Four-parameter logistic (4PL) regression was used to estimate the concentration of p24 in the plasma samples (accounting for the dilution factor) using the manufacturer’s software analysis. Wells were considered positive for the presence of p24 if the median concentration for three triplicates was above 0.0159 pg/mL. For testing sensitivity and specificity, we analyzed plasma samples from 82 HIV-negative individuals and 12 treatment-naïve PLWH (Supplementary Data 1).

### Assessment of integrated HIV DNA or cell-associated HIV RNA

Cryopreserved peripheral blood mononuclear cells (PBMCs) isolated from PLWH were thawed at 37 °C and transferred into complete RPMI medium (R10), consisting of RPMI 1640 supplemented with 10% heat-inactivated fetal calf serum, 2 mM L-glutamine, 100 U/mL penicillin, and 100 µg/mL streptomycin. Cells were rested overnight at 37 °C and 5% CO₂. The following day, CD4 T cells were isolated via magnetic negative selection using the CD4 T Cell Isolation Kit (Miltenyi Biotec). Genomic DNA (gDNA) was extracted from the purified CD4 T cells using the QIAamp DNA Mini Kit (Qiagen), following the manufacturer’s protocol. DNA purity and concentration were assessed using a NanoDrop spectrophotometer. Total RNA was extracted from the same CD4 T cell samples using QIAzol Lysis Reagent (Qiagen), followed by RNA purification per the manufacturer’s protocol. For the quantification of integrated HIV DNA (HIV-1 Integrase qPCR), isolated genomic DNA (gDNA) from CD4⁺ T cells was first pre-amplified using specific primers targeting HIV-1 integrase and CD3 (the latter used for normalization to cell input) (Supplementary Data 2). Amplification reactions were performed using 2× DreamTaq Master Mix (Thermo Fisher Scientific). For CD3 pre-amplification, the thermal cycling conditions were as follows: an initial step at 95 °C for 8 min, followed by 12 amplification cycles of 95 °C for 1 min, 55 °C for 40 s, and 72 °C for 1 min, and a final elongation step at 72 °C for 15 min. For HIV-1 integrase pre-amplification, the program was identical in structure but used extended elongation: 95 °C for 8 minutes, followed by 12 cycles of 95 °C for 1 min, 55 °C for 1 min, and 72 °C for 10 min, with a final elongation step at 72 °C for 15 min. Quantitative PCR (qPCR) was then performed on the pre-amplified DNA using HIV integrase- and CD3-specific primers and fluorescent probes on a Bio-Rad CFX384 Real-Time PCR System. The qPCR program consisted of an initial denaturation at 95 °C for 3 minutes, followed by 44 amplification cycles of 95 °C for 30 s, 60 °C for 30 s, and 72 °C for 1 minute. For the quantification of cell-associated HIV RNA (ALU qPCR), total RNA isolated from CD4 T cells was reverse-transcribed to generate HIV-specific cDNA using ULF1 and UR1 primers, 2× DreamTaq (Thermo Fisher Scientific), and SuperScript III Reverse Transcriptase (Thermo Fisher Scientific). The reverse transcription protocol included an incubation at 50 °C for 30 minutes, followed by a denaturation step at 94 °C for 2 min, and 16 amplification cycles of 94 °C for 15 s, 55 °C for 30 s, and 68 °C for 1 minute. A final elongation was performed at 68 °C for 5 min. The resulting cDNA was pre-amplified using ULF1, ALU1, and ALU2 primers with 2× DreamTaq. The cycling conditions were 95 °C for 8 minutes, followed by 12 cycles of 95 °C for 1 min, 55 °C for 1 min, and 72 °C for 10 min, with a final elongation at 72 °C for 15 min. For qPCR, the pre-amplified cDNA was amplified using Lambda T and UR2 primers together with an HIV-specific fluorescent probe. Reactions were run on the Bio-Rad CFX384 system using the following protocol: 95 °C for 3 min, followed by 44 cycles of 95 °C for 30 s, 60 °C for 30 s, and 72 °C for 1 min.

### Assessment of T cell activation profile

Cryopreserved PBMC samples were thawed at 37 °C and transferred to R10 media. Cells were then centrifuged for 10 min at 300 × g, and the supernatant was decanted. This washing step was repeated two times, after which the cells rested overnight at 37 °C and 5% CO_2_. The next morning, PBMCs were counted, seeded in 96-well U-bottom plates at a density of 1 million/well and washed with PBS. Subsequently, cells stained for viability in 100 µl of 1% solution of ZombieAqua dye (Biolegend; 423102) in PBS for 15 min at 4 °C. Next, samples were washed with FACS buffer (PBS supplemented with 2% FCS, 0.05% NaN_3_, and 2 mM EDTA), and stained extracellularly for 15 min at 4 °C with the fluorescently conjugated antibodies: anti-CD3-APC-Cy7 (clone UCHT1; Biolegend; 300426; 1:80), anti-CD4-BV786 (clone RPA-T4; Biolegend; 300554; 1:40), anti-CD8a-AF700 (clone HIT8a, Biolegend; 301028; 1:40), anti-PD-1-PE-Cy7 (clone A17188B, Biolegend; 621616; 1:20), anti-CD38-BV605 (clone HB-7, Biolegend; 356642; 1:40), and anti-HLA-DR-APC (clone LN3, Biolegend; 327022; 1:40). Subsequently, samples were washed once with FACS buffer, twice with PBS and acquired on a BD FACS Celesta with BD FACSDiva™ Software Version 8.0 (BD Bioscience). All antibodies were checked for performance and titrated before use. The frequencies of activation were determined based on fluorescence minus one (FMO) controls. Possible longitudinal fluctuations in laser intensity were monitored before every experiment using fluorescent beads (Rainbow beads, Biolegend). PMT voltages were adjusted accordingly to ensure constant signal intensity over time. The data were analyzed using the FlowJo Software version 10.10.0 (TreeStar).

### Assessment of T cell cytokine expression profile

Cryopreserved PBMC samples were thawed at 37 °C and transferred to R10 media. Cells were then centrifuged for 10 min at 300 × g, and the supernatant was decanted. This washing step was repeated two times, after which the cells were rested overnight at 37 °C and 5% CO_2_. The next morning, PBMCs were counted, seeded in 96-well U-bottom plates at a density of 1 million/well and stimulated with a HIV-1 Gag peptide pool of 150 overlapping 15mers peptides (JPT), a co-stimulatory anti-CD28/CD49d antibody (BD Bioscience; 347690; 1:100) and an anti-CD107a-Pe/Dazzle antibody (clone H4A3; Biolegend; 328646; 1:40) for 6 h at 37 °C. GolgiStop and GolgiPlug (BD Bioscience) were added 1 h into the stimulation. An equally treated negative control without the peptides was included for each sample. A positive control where cells were stimulated with PMA (20 ng/ml) (Sigma-Aldrich) and ionomycin (1 μg/ml) (Sigma-Aldrich) was included for each experiment. Following stimulation, cells were washed with PBS and stained for viability in 100 µl of 1% solution of ZombieAqua dye (Biolegend; 423102) in PBS for 15 min at 4 °C.  Next, samples were washed with FACS buffer (PBS supplemented with 2% FCS, 0.05% NaN_3_, and 2 mM EDTA), and stained extracellularly for 15 min at 4 °C with the fluorescently conjugated antibody anti-CD8a-AF700 (clone HIT8a, Biolegend; 300920; 1:40). Subsequently, samples were washed with FACS buffer, fixed, and permeabilized in CytoFix/CytoPerm Solution (BD Bioscience) for 15 min at 4 °C. Fixed cells were washed with 1x Perm/Wash Buffer (BD Bioscience), and stained intracellularly with fluorescently conjugated antibodies: anti-CD3-APC-Cy7 (clone UCHT1; Biolegend; 300426; 1:40), anti-CD4-BV786 (clone RPA-T4; Biolegend; 300554; 1:20), anti-IFN-γ-PE (clone B27; Biolegend; 506507; 1:40) and anti-TNF-α-BV605 (clone MAb11; Biolegend; 502936; 1:20) for 15 min at 4 °C. After incubation, cells were washed once with 1x Perm/Wash Buffer, twice with PBS, and acquired on a BD FACS Celesta with BD FACSDiva™ Software Version 8.0 (BD Bioscience). All antibodies were previously established and titrated to optimal concentrations. The frequencies of antigen-specific T cells were calculated as negative-control-subtracted data. Possible longitudinal fluctuations in laser intensity were monitored before every experiment using fluorescent beads (Rainbow beads, Biolegend). PMT voltages were adjusted accordingly to ensure constant signal intensity over time. The data were analyzed using the FlowJo Software version 10.10.0 (TreeStar).

### Statistical analysis

To assess whether the strength of the correlation between plasma p24 levels and viral load changed significantly between Day 0/3 and Day 84 within the same individuals, we used Steiger’s Z-test for comparing two dependent correlation coefficients^49^. This test accounts for the non-independence of repeated measurements from the same individuals. Spearman correlation coefficients were calculated using Prism (GraphPad Software) for p24 and viral load at both time points: Day 0/3: r₁ = 0.83 and Day 84: r₂ = 0.20. To correct for the dependence between repeated measures, we calculated the correlations between the same variables across time: Spearman correlation coefficient between p24 levels at Day 0/3 and Day 84: rₓ = 0.31 as well as correlation coefficient between viral load at Day 0/3 and Day 84: rᵧ = 0.28. These values were used to compute the Steiger’s Z statistic using the following formula **(1)** (with *n* = 36):

1 $$Z=\frac{r1-r2}{\sqrt{\frac{2(1-{ravg})}{n-3}}}{with\; r}{\rm{\_}}\_{avg}=({r}_{{\rm{x}}}+{r}_{{\rm{\gamma }}})/2$$

Statistical analysis was performed using Prism (GraphPad Software) and RStudio (2022.07.0). Differences between groups were assessed using the Mann-Whitney test for unpaired data and the strength of correlations was evaluated by Spearman’s test. Statistical differences between time points were assessed using a minimal assumptions two-sided sign test, which takes into account that values below the LOD are imputed. Statistical significance is indicated by the following annotations: *p < 0.05, **p < 0.01, ***p < 0.001, ****p < 0.0001.

## Results

### Detection of ultra-low levels of p24

**Fig. 1:**
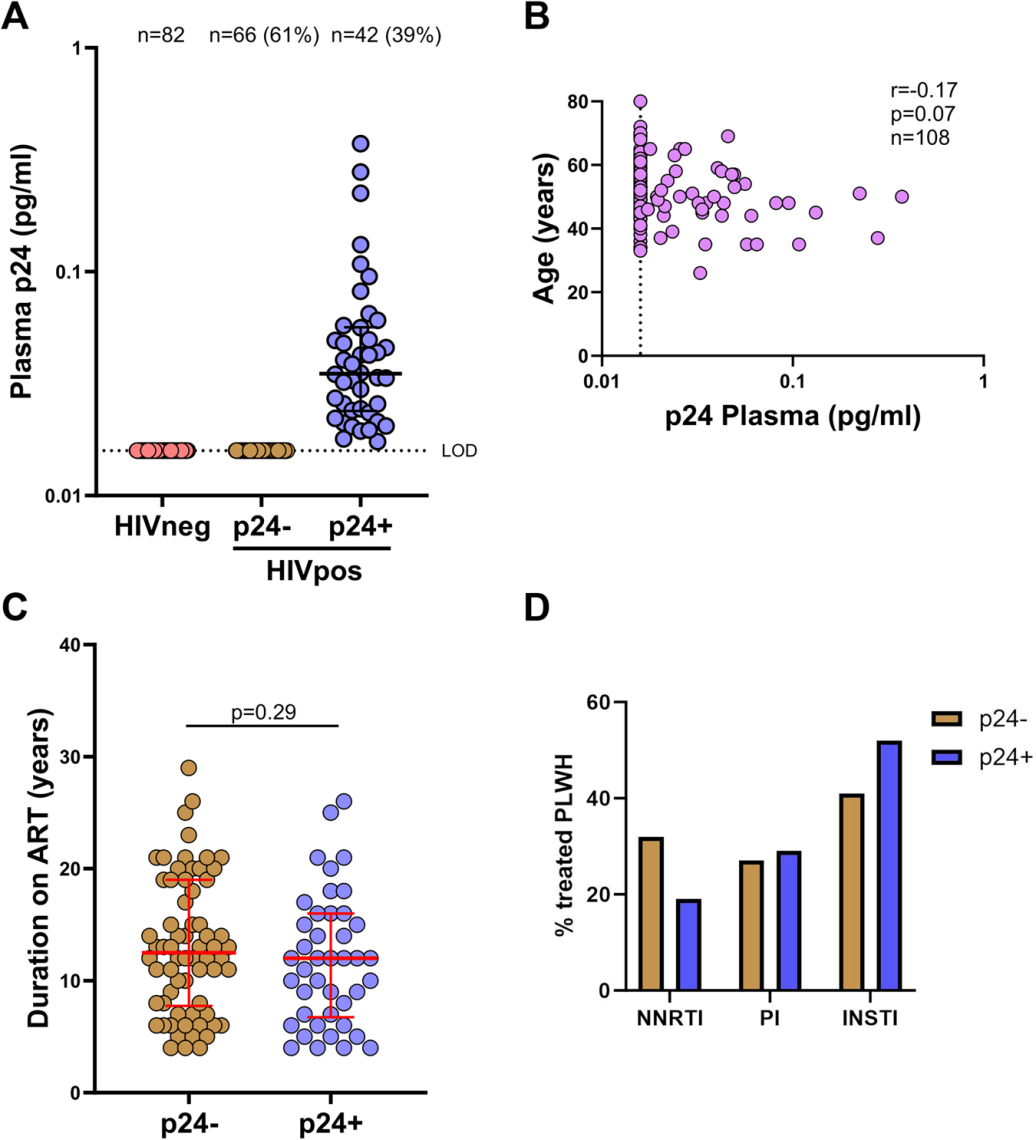
Detection of ultra-low levels of p24. **A** Plasma p24 levels were measured in donors without HIV-1 (*n* = 82, peach-colored circles) and in treated PLWH (*n* = 108) who maintained HIV RNA levels below the detection limit (<30 copies/mL) for over four years. The dashed line represents the assay’s limit of detection (LOD), set at 16 fg/mL. Values below the LOD were assigned an arbitrary value of 15.9 fg/mL. Error bars represent the median value with interquartile range (IQR). Light brown circles denote PLWH without detectable p24 (*n* = 66), and violet circles denote PWLH with detectable p24 (*n* = 42). The Y-axis is displayed on a logarithmic scale. **B** Age of treated PLWH (*n* = 108) was correlated with plasma p24 levels. The dashed line represents the p24 assay LOD, set at 16 fg/mL. The strength and significance of the correlation between viral load and p24 levels were assessed using a two-sided nonparametric Spearman’s rank correlation test. **C** Duration on ART was compared between PLWH without detectable p24 (p24-; brown circles; *n* = 66) and those with detectable p24 (p24 + ; violet circles; *n* = 42). Error bars represent the median with interquartile range (IQR). Statistical significance was assessed using the two-sided Mann-Whitney test for unpaired data. **D** Percentage of treated PLWH with and without detectable p24, divided by ART regimen at the time of sampling. Individuals without detectable p24 (p24 − ; *n* = 66) are shown in brown bars, and those with detectable p24 (p24 + ; *n* = 42) are shown in violet bars.

To determine residual viral protein production in treated PLWH, we optimized a digital ELISA using Simoa® technology, enabling detection of ultra-low p24 levels in femtomolar concentrations. Regular and ultrasensitive HIV-1 p24 ELISAs have a detection limit of 5 pg/ml. By utilizing microscopic beads coated with anti-p24 antibodies, the assay improves p24 detection limits by 1000-fold compared to conventional ELISA. To assess the limitations of this ultrasensitive assay, we first established our own robust limit of detection (LOD) for p24 measurement. We analyzed plasma of 82 donors without HIV-1. The manufacturer specifies a LOD of 0.0045 pg/ml (4.5 fg/ml) and a lower limit of quantification (LLOQ) of 0.0160  pg/ml (16 fg/ml). Out of the 82 donors without HIV-1, we did not detect p24 levels above 16 fg/mL in any of the plasma samples (Fig. 1A). Based on these results, we set a LOD with a cutoff of 16 fg/mL for our analyses and assigned an arbitrary value of 15.9 fg/mL for p24 levels below this LOD.

To assess residual viral protein production, we measured plasma p24 at ultra-low concentrations in 108 treated PLWH (chronic cohort) who had maintained HIV-1 RNA levels below the detection limit (<30 copies/mL) for over four years. We detected ultra-low p24 levels ranging from 17 to 370 fg/mL in 42 of 108 individuals (39%) (Fig. 1A). Interestingly, ultra-low p24 levels were remarkably stable in treated PLWH when measured at two different time points 6 months apart (*n* = 37) (Supplemental Fig. 1). There was also no significant correlation with residual p24 levels and demographic or clinical markers (Supplementary Table 1; Fig. 1B) including duration on therapy (p24 + : 13 years [4–26]; p24-: 12 years [4–29]; *p* = 0.29) (Fig. 1C). Notably, prior CMV infections (*n* = 7) were observed exclusively in treated PLWH without detectable low-level p24. Additionally, we analyzed the ART regimen of treated PLWH at the time of sampling (Supplementary Table 2), but no difference was observed between the groups with and without detectable p24 (*p* = 0.40) (Fig. 1D). Furthermore, no significant correlation was observed between plasma p24 levels and either integrated HIV DNA or cell-associated HIV RNA (*n* = 27) (Supplementary Fig. 2).

Taken together, ultra-low level of p24 production are detectable in a non-definable subset of PLWH that are on long-term successful antiretroviral therapy for at least 4 years.

### Reduction in p24 levels following dual PI therapy, but not two-drug regimen switch

**Fig. 2:**
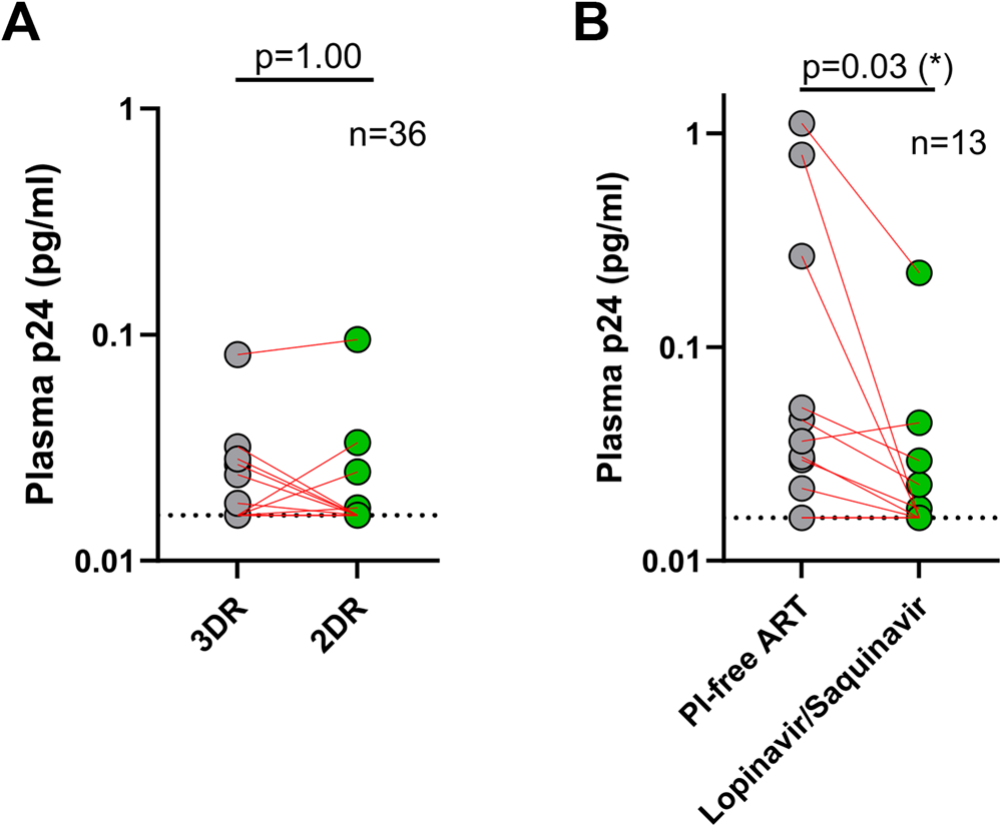
Reduction in p24 levels following dual PI therapy but not 2DR switch. **A** Plasma p24 levels were measured in treated PLWH (*n* = 36) who switched from a triple-drug regimen (3DR) to a dual regimen with either dolutegravir/lamivudine (*n* = 22) or dolutegravir/rilpivirine (*n* = 14). p24 levels were assessed for each individual before the switch (grey circles) and again after 12 or 24 months (green circles). Number of patients with p24 values above the assay’s LOD: 3DR: 6/36 (17%) and 2DR: 4/36 (11%). Statistical difference between time points were assessed using a two-sided sign test. **B** Plasma p24 levels were measured in treated PLWH (*n* = 13) who switched from a Protease inhibitor-(PI)-free ART regimen to a dual protease inhibitor treatment with lopinavir/saquinavir. Grey circles represent p24 levels before the switch for each individual, and green circles represent levels after the switch. Number of patients with p24 values above the assay’s LOD: PI-free ART: 9/13 (69%) and Lopinavir/Saquinavir: 5/13 (385%). Statistical difference between time points were assessed using a two-sided sign test. *P*-value  <  0.05 (*) was considered to be significant. The Y-axes are displayed on a logarithmic scale.

We next wanted to determine whether different therapeutic strategies may influence residual p24 levels. We first investigated a switch from a three-drug regimen (3DR) to a two-drug regimen (2DR). We analyzed a cohort of 36 PLWH who switched from 3DR therapy to a dual regimen with either dolutegravir/lamivudine (*n* = 22) or dolutegravir/rilpivirine (*n* = 14). p24 levels were assessed before the switch and again after 12 or 24 months. However, no significant change in low-level p24 production was observed (*p* = 1.00) (Fig. 2A). Next, we examined the impact of PI-based therapy, which targets the last stage of the HIV life cycle - virion assembly - compared to integrase strand transfer inhibitors (INSTIs) or non-nucleoside reverse transcriptase inhibitors (NNRTIs), which act before viral integration. We hypothesized that PIs, by allowing partial viral protein production prior to blocking maturation, might result in increased p24 levels but not HIV viremia. To test that, we analyzed a cohort of 13 PLWH with a mean age of 42 years [36–45] *(*Supplementary Table 3), who switched from a PI-free ART regimen to a dual PI treatment with lopinavir/saquinavir. At baseline, under PI-free ART, ultra-low p24 levels were detectable in 69% (9 out of 13) of participants, with a median concentration of 45 fg/ml [21–1120 fg/ml] calculated excluding imputed values below the LOD (Fig. 2B). Surprisingly, following the switch to dual PI therapy, p24 levels declined significantly (*p* = 0.03). At the 6-month follow-up, only 38% of participants (5 out of 13) still had detectable p24 (Supplementary Data 3). Among these individuals, the median p24 concentration was 29 fg/ml [17–44 fg/ml]. Unfortunately, PBMC samples were not available from this cohort, limiting our ability to analyze potential immunological mechanisms, such as increased CD8 T cell activity, that might contribute to this reduction in residual p24 levels.

In summary, while dual PI therapy led to a significant reduction in p24 levels, switching from a 3DR to a 2DR regimen did not have any impact on p24 levels.

### Levels of p24 dropped upon ART initiation but remained detectable in 20% of chronic PLWH

**Fig. 3:**
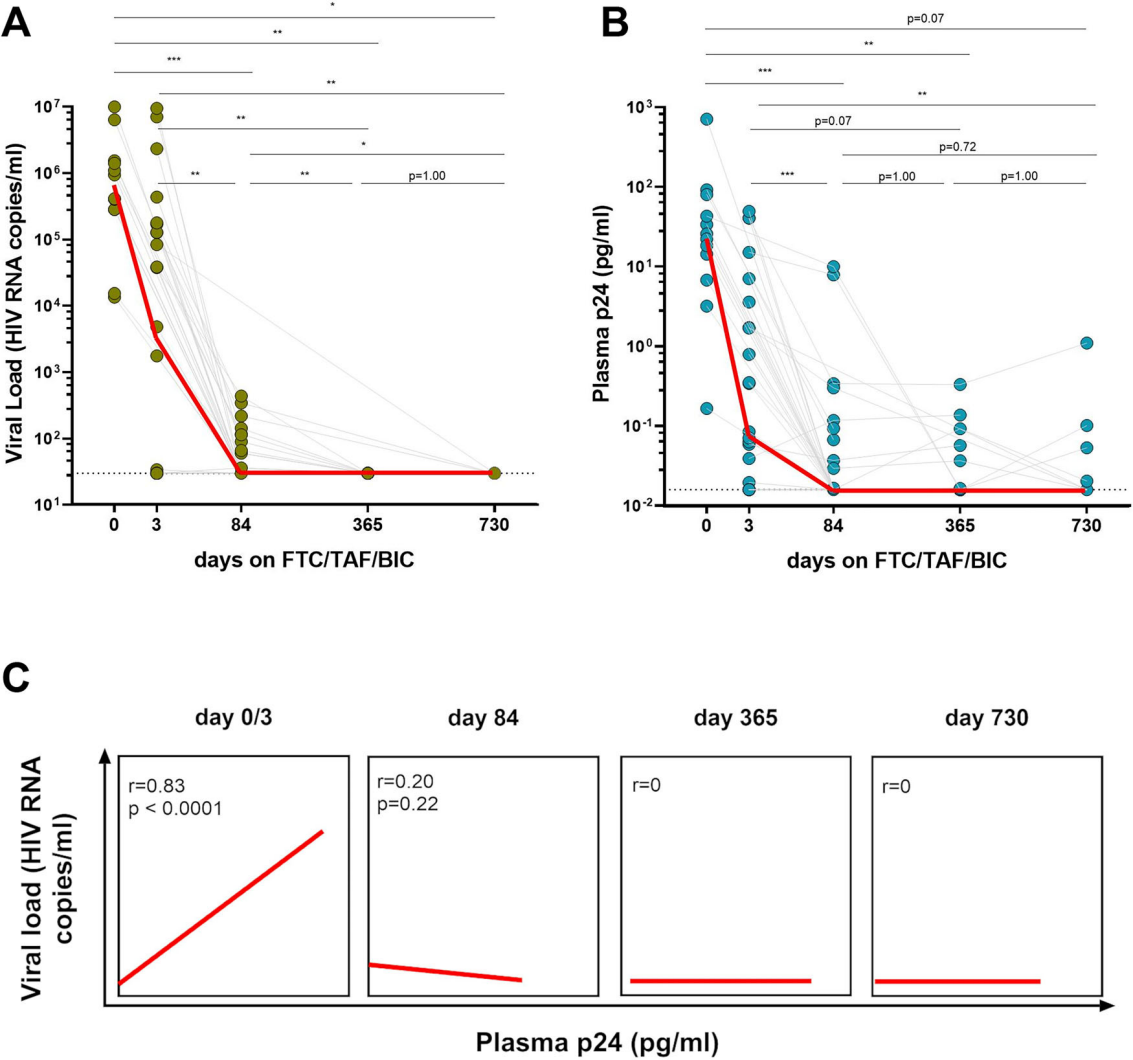
Levels of p24 dropped upon ART initiation but remained detectable in 20% of chronic PLWH. A cohort of 41 individuals who initiated suppressive ART (FTC/TAF/BIC treatment) during acute HIV and remained virally suppressed were analyzed over a 2-year period. **A** Viral load was measured during the acute infection (day 0 OR day 3 after treatment initiation) as well as day 84, 365, and 730 after treatment initiation. The dashed line represents the assay’s LOD: <30 HIV RNA copies/ml for viral load. Number of patients with viral load above the assay’s LOD: Day 0: 12/12 (100%); Day 3: 14/24 (58%); Day 84: 12/40 (30%); Day 365: 0/31 (0%); Day 730: 0/20 (0%). The red lines connect the median values of each time point. Statistical difference between time points were assessed using a two-sided sign test. The Y-axis is displayed on a logarithmic scale. **B** p24 levels were measured during the acute infection (day 0 OR day 3 after treatment initiation) as well as day 84, 365, and 730 after treatment initiation. The dashed line represents the assay’s LOD: 16 fg/mL p24. Number of patients with p24 values above the assay’s LOD: Day 0: 12/12 (100%); Day 3: 17/24 (71%); Day 84: 10/40 (25%); Day 365: 6/31 (19%); Day 730: 4/20 (20%). The red lines connect the median values of each time point. Statistical difference between time points were assessed using a two-sided sign test. The Y-axis is displayed on a logarithmic scale. **C** Correlation of viral load and plasma p24 levels at multiple time points following ART initiation (days 0/3, 84, 365, and 730). Red lines indicate linear regression fits for visualization purposes. The strength and significance of the associations were assessed using a two-sided nonparametric Spearman’s rank correlation test with correlation coefficients (r) and *p*-values reported. *P*-values < 0.05 (*),  < 0.01 (**), < 0.001 (***), or < 0.0001 (****) were considered to be significant.

As HIV viremia and HIV protein production do not correlate, we next assessed whether this is true during all stages of HIV. We therefore studied HIV p24 concentration longitudinally before and after ART initiation as well as from the acute to chronic phases of HIV. We used a cohort (RV464) of 41 individuals who initiated suppressive ART (Emtricitabine (FTC) /Tenofovir Alafenamide (TAF) /Bictegravir (BIC)) during acute HIV and remained virally suppressed over a 2-year period. We measured viral load and p24 levels during acute HIV (day 0 OR day 3 after treatment initiation) as well as at day 84, 365, and 730 after treatment initiation. As expected, viral load dropped below the LOD (<30 HIV-1 RNA copies/ml) for all participants by day 365 and remained fully virologically suppressed for up to two years into the chronic phase of the infection (Fig. 3A). Similar to the reduction in HIV-1 RNA, we observed a significant reduction of plasma p24 levels from day 0 OR 3 (day 0/3) to day 84 (*p* < 0.001; *p* < 0.001 respectively), day 365 (*p* < 0.01; *p* = 0.07 respectively) and day 730 (*p* = 0.07; *p* < 0.01 respectively) after treatment initiation (Fig. 3B, Supplementary Data 3). Despite HIV-1 RNA being undetectable one and two years after ART initiation, p24 protein remained detectable in 19% of participants (6/31; range: 16.4-331.8 fg/mL) at one year and 20% (4/20; range: 20.4–1100.4 fg/mL) at two years. We next compared the correlation coefficients of p24 levels and viral load between day 0/3 and day 84 and observed a Steiger Z-score of 3.05, indicating a statistically significant difference (p < 0.01) between the two time points. At day 0/3, the Spearman’s correlation coefficient (r) was 0.83 (*p* < 0.0001), indicating a strong and significant correlation between p24 and HIV RNA levels shortly after ART initiation. By contrast, at day 84, the correlation was strikingly weaker and no longer significant (*r* = 0.20, *p* = 0.21) (Fig. 3C). These findings indicate that while p24 levels closely reflect viral RNA early in treatment, this association diminishes significantly by day 84.

In summary, p24 levels decreased significantly following ART initiation, transitioning from acute to chronic infection, but remained detectable at ultra-low levels in 20% of participants over a 2-year period.

### Individuals with detectable p24 levels show higher levels of T cell activation

**Fig. 4:**
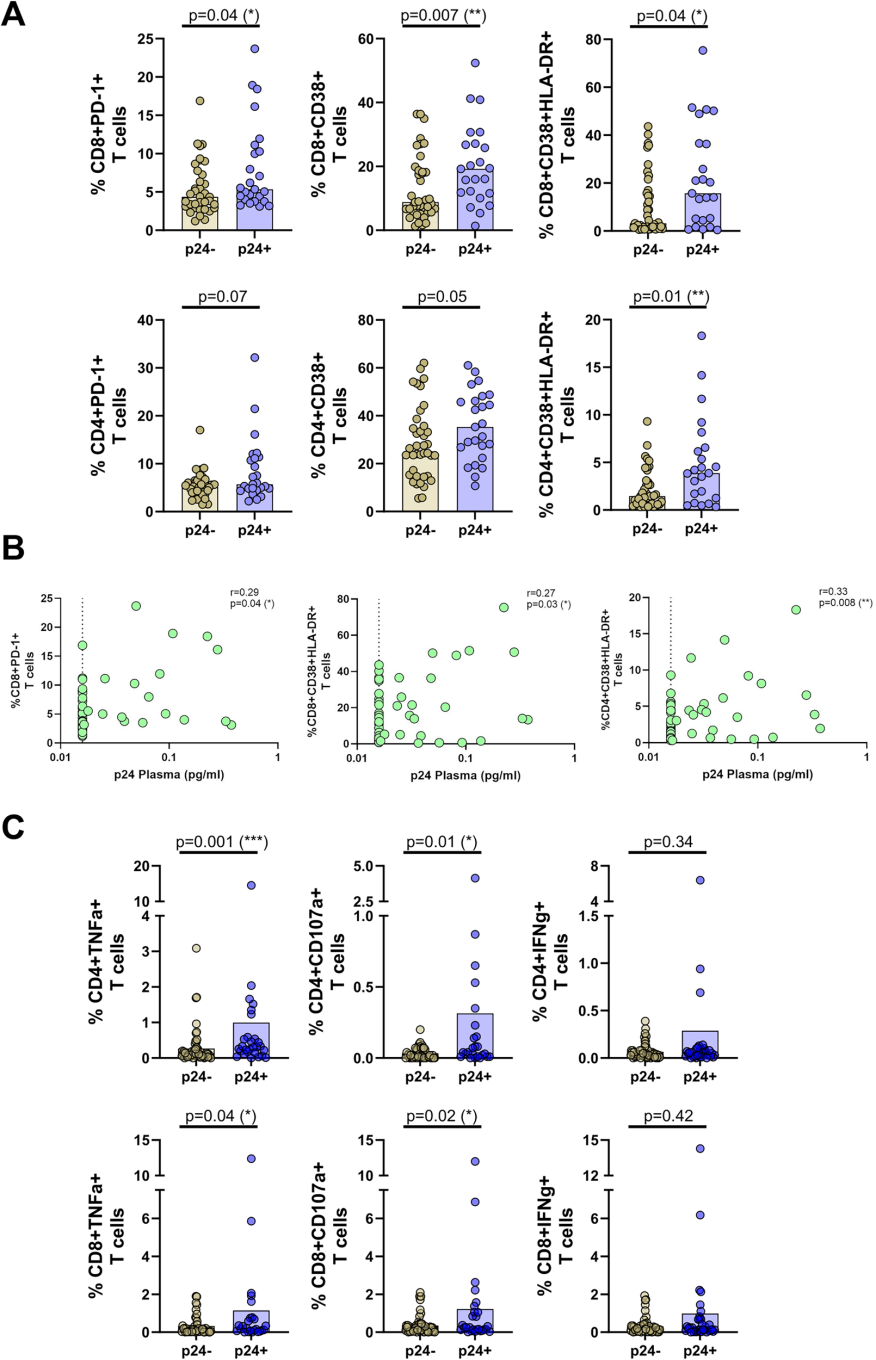
Individuals with detectable p24 levels show higher levels of T cell activation. **A** To analyze inhibitory and activation markers on CD8 (upper panel) and CD4 (lower panel) T cells, PBMCs were isolated from a total of 88 treated PLWH from our chronic and RV464 cohort, either without (p24-; dark yellow circles) or with (p24 + ; violet circles) detectable plasma p24. Cells were stained for CD38 (*n* = 64), HLA-DR (*n* = 64), and PD-1 (*n* = 61). Notably, CD8 and CD4 T cells were analyzed directly ex vivo without additional stimulation. Graphs show the percentage of CD8 or CD4 T cells expressing CD38, CD38 + HLA-DR, or PD-1. Horizontal bars indicate median values. Statistical significance was assessed using the two-sided Mann-Whitney test for unpaired data. **B** The percentage of CD8 T cells expressing PD-1 (left graph), CD38 + HLA-DR (middle graph), and CD4 T cells expressing CD38 + HLA-DR (right graph) were correlated with plasma p24 levels. The dashed line represents the p24 assay LOD, set at 16 fg/mL. The strength and significance of the correlation were assessed using a two-sided nonparametric Spearman’s rank correlation test. *P*-values  <  0.05 were considered statistically significant. Spearman’s r > 0 indicates a positive correlation between the variables. The X-axis is displayed on a logarithmic scale. **C** To analyze anti-HIV-specific immune responses for CD4 (upper panel) and CD8 (lower panel) T cells, PBMCs were isolated from 93 treated PLWH, either without (p24-; dark yellow circles) or with (p24 + ; violet circles) detectable plasma p24 and stimulated with a Gag peptide pool for 6 h. Cells were stained for CD107a, IFN-γ, and TNF-α, and graphs show the percentage of CD8 or CD4 T cells expressing these functional markers. Horizontal bars indicate median values. Statistical significance was assessed using the two-sided Mann-Whitney test for unpaired data. *P* values  <  0.05 (*),  <  0.01 (**) or < 0.001 (***) were considered to be significant.

We hypothesized that ongoing p24 production may be one of the main contributors to low-level immune activation. We therefore first analyzed inhibitory and activation markers on CD4 and CD8 T cells. PBMCs were isolated from 88 fully virologically suppressed PLWH from our chronic and RV464 cohort, with or without detectable p24, and stained for CD38, HLA-DR, and PD-1 (Supplementary Table 4 and Supplementary Fig. 3*)*. Notably, CD4 and CD8 T cells were analyzed directly ex vivo without additional stimulation. Interestingly, we observed a significantly higher percentage of CD8 T cells expressing the inhibitory marker PD-1 (*p* < 0.05), the activation marker CD38 (*p* < 0.01), as well as dual expression of CD38 and HLA-DR (*p* < 0.05) in individuals with detectable p24 compared to those without detectable p24 (Fig. 4A). In CD4 T cells, there was a significantly higher expression of CD38 together with HLA-DR (*p* = 0.01) in individuals with detectable p24 compared to those without. PD-1 expression showed no significant difference (*p* = 0.07). The activation status of CD8 and CD4 T cells positively correlated with ultra-low levels of p24. We found that the percentage of CD8 and CD4 T cells expressing CD38 and HLA-DR significantly (*p* < 0.05; *p* < 0.01, respectively) correlated with the amount of detectable p24 (Fig. 4B). Similarly, the expression of the inhibitory marker PD-1 on CD8 T cells also showed a significant positive correlation with p24 levels (*p* < 0.05).

Given the elevated activation status, we next assessed whether treated PLWH exhibited an enhanced anti-HIV-specific immune response. To investigate this, PBMCs were isolated from fully virologically suppressed PLWH (*n* = 93) undergoing ART and stimulated with a Gag peptide pool (Supplementary Table 5 and Supplementary Fig. 4*)*. The HIV-specific response was assessed using multicolor flow cytometry and a polyfunctionality panel that included three functional markers: CD107a, IFN-γ, and TNF-α. Although all individuals were fully virologically suppressed, we observed that CD4 and CD8 T cells from individuals with detectable p24 in plasma expressed significantly higher amount of TNF-α (*p* < 0.01; *p* < 0.05 respectively) and CD107a (*p* = 0.01; *p* < 0.05 respectively) compared to individuals without detectable p24 (Fig. 4C). A similar, but not statistically significant, trend was observed for IFN-γ in CD4 and CD8 T cells.

Taken together, the data suggest that individuals with ultra-low p24 levels have a more activated and robust HIV-specific T cell response despite ongoing treatment, indicating that they are still able to recognize HIV infected cells.

## Discussion

Although ART is highly effective at inhibiting HIV replication, it is not curative. In recent years, questions have been raised regarding the extent to which HIV replicates during ART and whether the observed proviruses are defective or intact^41,50,51^. Factors such as limited drug penetration within tissues^52,53^, decreased CD8 T cell effector functions under certain ART regimens^54^, and the presence of immune sanctuaries, such as B-cell follicles^55^, have been proposed as potential mechanisms enabling low-level HIV replication during ART. Most assays assess HIV persistence primarily by detecting HIV-1 RNA or reverse-transcribed DNA. While these assays are effective for assessing viral persistence and facilitating detailed analyses like sequence or integration diversity, they are limited in their ability to evaluate translationally competent reservoirs^56–58^. Until recently, the development and application of assays capable of detecting translation-competent viruses have been restricted by limits of detection that are far less sensitive than nucleic acid-based assays. We therefore have optimized a digital ELISA with the single-molecule array technology that allows the measuring of HIV Gag protein with sensitivity comparable to that of nucleic acid-based assays. We were able to detect ultra-low levels of p24 production despite the fact that all 108 tested PLWH were on successful ART for at least 4 years. Surprisingly, while dual PI therapy significantly reduced p24 levels, switching from a 3DR to a 2DR regimen did not affect p24 levels. Additionally, in another cohort, we observed that p24 levels decreased significantly following ART initiation, transitioning from the acute to chronic phase of HIV under ART, but remained detectable at ultra-low levels in 20% of participants over a 2-year period. Interestingly, p24 levels closely reflected viral RNA early in treatment; this association diminishes significantly already 3 months after ART initiation. Furthermore, we were able to demonstrate that virally suppressed PLWH with ultra-low p24 levels have a more activated and robust HIV-specific T cell response despite ongoing treatment.

We successfully applied a digital p24 readout to determine residual viral protein production in ART-treated PLWH. Compared to other studies^43,47,59^, we achieved p24 detection with a cut-off sensitivity of 16 fg/ml. This was made possible through key optimization steps, including sample preparation, specific dissociation of the antigen-antibody complexes, and an in-depth analysis of 82 HIV-1-negative samples. To the best of our knowledge, our results represent the first demonstration of ultra-low levels of p24 in a large cohort of long-term ART-treated PLWH, as well as insights into p24 behavior during acute versus chronic infection. Notably, we found no consistent demographic or clinical marker to explain residual p24 levels in approximately 40% of successfully treated PLWH. Unfortunately, we were unable to detect intracellular p24 in peripheral blood CD4 T cells from ART-treated PLWH using flow cytometry. This is technically very challenging, particularly without prior stimulation, due to the extremely low frequency of productively infected CD4 T cells in peripheral blood under suppressive therapy. This limitation highlights the importance of using highly sensitive plasma-based assays - such as the one employed in our study - to detect ultra-low levels of p24 that more accurately reflect the in vivo state at the time of sample collection.

Interestingly, we observed a difference in the proportion of treated PLWH with residual p24 between the long-term ART cohort and the cohort transitioning from acute to chronic infection. While the underlying cause is not fully understood, several key differences between the cohorts may explain this observation. Participants in the second cohort initiated ART shortly after HIV infection, likely limiting reservoir formation. In contrast, delayed ART initiation in the long-term cohort may have allowed for more extensive establishment of the viral reservoir or greater clonal expansion^60,61^. Additionally, treatment regimens differed: all individuals in the second cohort received bictegravir-based ART, which may influence residual antigen expression through differences in pharmacodynamics or tissue penetration^62^. Lastly, the long-term cohort had a longer duration of ART (≥4 years vs. 2 years), raising the possibility that persistent or even increasing p24 production over time could be linked to factors such as ongoing low-level transcription from the latent reservoir.

While high viral load at therapy initiation has been associated with a lower likelihood of achieving virological suppression in the past^63^, we observed no correlation between detectable p24 levels and viral load at therapy initiation. Similarly, higher baseline p24 levels were not predictive of residual p24 detection during long-term ART. Previous studies have demonstrated a strong correlation between plasma p24 antigen levels and viral RNA load, with reductions in p24 levels closely tracking decreases in viral load during effective ART^64^. Consistent with these findings, we observed a similar correlation, where p24 antigen levels closely mirrored viral RNA levels at therapy start. However, by 3 months into therapy, we could already see a significant shift in this relationship over time, resulting in a loss of correlation between the two markers. Of note, imputation of values below the LOD (to 15.9 fg/mL) may limit statistical power, which could contribute to the observed weakening of the correlation with viral load at Day 84 after ART initiation. Further studies have to understand why the association between RNA and p24 changes so quickly after ART initiation and how this affects low-level p24 production.

A critical question remains as to why ongoing protein production persists in individuals on suppressive ART. In recent years, more studies have explored the impact of replication-competent reservoirs, as well as the capability of defective and intact proviruses to express HIV proteins and contribute to persistent viral replication despite effective ART. Previous reports have emphasized the importance of characterizing cellular reservoirs that harbor proviruses capable of transcribing viral mRNAs (termed transcription-competent) and translating viral proteins (translation-competent)^51,57,65–67^. Notably, it has been shown that p24 protein expression, although rare, can be observed in stimulation-induced viral RNA-positive (vRNA + ) cells from ART-suppressed individuals^66^. HIV integration into transcriptionally active regions of the host genome can lead to sporadic or consistent HIV RNA transcription, even in the absence of full viral replication. These transcriptional bursts may result in the production of viral proteins, including the p24 antigen^57,68,69^. Such transcriptional activity is often influenced by factors such as the local chromatin environment^68^, cellular activation states, or host signaling pathways^70^. Another plausible mechanism for the detected p24 antigen is “cellular shedding”, whereby destroyed cells release viral proteins or RNA. This may explain the presence of free p24 antigen in plasma, even when replication of intact proviruses is not evident. However, results from a recent study using the digital p24 assay in Q-VOA experiments suggest the production of properly assembled virus particles rather than the release of unassembled capsid from dying cells^47^. It is important to note that common PCR-based techniques, including those that measure total and integrated HIV DNA, tend to vastly overestimate the size of the HIV reservoir due to the high prevalence of integrated but defective proviruses^67^. Additionally, Q-VOA may underestimate the reservoir size, as not all replication-competent proviruses can be induced by a single round of stimulation^71^. Our data suggest that the use of Simoa® as an ultrasensitive p24 detection method, combined with complementary techniques to measure persistent HIV reservoirs, can provide a more comprehensive assessment of latent viral burden and low-level viral production under ART. Another possibility is that low drug penetration of certain ART regimens, leading to persistent HIV reservoirs, contributes to low-level p24 production^53,72,73^. However, we observed no major differences in commonly used ART regimens between individuals with and without detectable p24, suggesting that reduced drug activity may not fully explain residual p24 levels. Nevertheless, our findings suggest that specific modifications in the ART strategy can influence residual p24 production. While a change from a three-drug to a two-drug regimen did not significantly impact p24 production, the unexpected decline in p24 following initiation of dual PI therapy is particularly intriguing. Given that protease inhibitors act at a late stage of the HIV replication cycle - after viral protein synthesis but before virion maturation - we initially hypothesized that incomplete inhibition might allow for accumulation of viral proteins such as p24^74–76^. However, the observed decrease suggests additional factors at play, potentially involving enhanced immune-mediated clearance or improved suppression of viral protein expression. That being said, this effect appears limited to a dual-PI-only regimen, as we did not observe differences in p24 levels among PLWH receiving a PI as part of a standard three-drug regimen. The lack of available PBMCs from this cohort limits mechanistic insight, but future studies may clarify whether enhanced CD8 T cell function or changes in cellular reservoirs contribute to this effect^77,78^.

Similar to the persistent presence of HIV, residual immune activation has been observed even in individuals on successful ART. Studies have shown that T cells remain activated in PLWH undergoing treatment, even when the virus is undetectable^26,28–30^. The underlying causes of this chronic inflammation and immune activation in HIV infection under ART are not yet fully understood. Here, we demonstrated a direct correlation between treated PLWH with detectable p24 and a more activated, robust anti-HIV-specific immune response. Furthermore, we observed that higher p24 levels were associated with increased activation of both CD8 and CD4 T cells. It is known that the HLA class I-restricted CD8 T-cell responses contribute substantially to the control of HIV-1 replication in PLWH^79,80^. The appearance of HIV-specific CD8 T cells is temporally associated with the decline of plasma viremia after acute infection, and their presence is associated with the control of viral replication during chronic HIV^81,82^. However, after ART initiation CD8 counts normalize while T cell activation and exhaustion decrease compared to untreated PLWH. Nevertheless, in many cases, the subsets and activation status of CD8 T cells do not fully normalize compared to people without HIV^25–33^. The mechanisms underlying these CD8 persistence remain largely unknown, which may include bystander activation, exhaustion, or recognition of defective provirus^38^. We propose that, among other factors, persistent low-level p24 production may serve as a driver of residual immune activation in treated PLWH. Furthermore, it is possible that even minimal HIV protein production acts as a critical catalyst to increase anti-HIV-specific immunity of CD8 T cells. It has been demonstrated that in some individuals, Gag-specific CD8 T cell responses are present despite the absence of detectable HIV replication^83^. CD8 T cells can durably suppress viral replication as demonstrated by spontaneous HIV controllers, and it has been proposed that CD8 T cells contribute to viral suppression during chronic infection under ART^84^. Some PLWH continue to exhibit detectable immune responses even after years of effective ART. Specifically, PLWH who have achieved successful treatment often display elevated levels of inflammatory markers such as IL-6 or sCD14 when compared to other treated PLWH^85^. Among various potential factors, continuous exposure to residual viral antigens could contribute to increased antigen-specific CD8 T cell responses. Although this can lead to immune exhaustion, an alternative scenario is conceivable in which the CD8 T cell response, caused by residual p24 levels, may be capable of reducing the viral reservoir.

In conclusion, we were able to detect ultra-low levels of p24 in ART-treated virally-suppressed PLWH, suggesting persistent low-level p24 production may serve as a driver of residual immune activation in treated PLWH. Furthermore, we demonstrated that an optimized Simoa® approach is an optimal method for ultrasensitive p24 detection. When combined with complementary techniques to measure persistent HIV reservoirs, this approach can offer a more comprehensive assessment of latent viral burden and low-level viral replication under ART. Further investigations are necessary to fully understand the mechanism of action involved in residual p24 production and persistent immune action.

## Supplementary information

SUPPLEMENTAL MATERIAL

Description of Additional Supplementary Files

Supplementary Data 1

Supplementary Data 2

Supplementary Data 3

Supplementary Data 4

Supplementary Data 5

Supplementary Data 6

Supplementary Data 7

Supplementary Data 8

## Data Availability

The source data for Fig. 1 is in Supplementary Data 4, for Fig. 2 is in Supplementary Data 5, for Fig. 3 is in Supplementary Data 6, for Fig. 4a, b is in Supplementary Data 7 and for Fig. 4c is in Supplementary Data 8. Data (which is not available in the source data) contains information that could compromise the privacy of research participants. Data sharing restrictions imposed by national and transnational data protection laws prohibit the general sharing of data. However, upon submission of a proposal to the corresponding author and approval of this proposal by (i) the principal investigator, (ii) the Ethics Committee of the University of Bonn, and (iii) the data protection officer of the University Hospital Bonn, data collected for the study can be made available to other researchers. Requests will be reviewed and responded to within six weeks of submission.
