# Supplementary material for: Ultra-low level HIV p24 drives immune activation in antiretroviral therapy-treated people living with HIV
Source: Commun Med (Lond). 2025 Dec 2;5:510. doi: 10.1038/s43856-025-01261-3 (PMC12672821; doi:10.1038/s43856-025-01261-3)
Supplement: Supplementary file 1 — SUPPLEMENTAL MATERIAL [file 43856_2025_1261_MOESM1_ESM.pdf]

Supplementary Information

Manuscript title: Ultra-low level HIV p24 drives immune activation in antiretroviral therapy-treated people living with HIV

Authors: Richter, Enrico et al.

Supplementary Table 1

treated PLWH				
**Demographics***	**Overall (n=108)**	p24+ (n=42)	p24- (n=66)	P-value
**Age**				0.12
Median [range]	50 [26 - 80]	49 [26 - 69]	51 [33 - 80]	
**Gender**				0.13
Male	94	33	60	
Female	15	9	6	
**Years on treatment**				0.29
Median [range]	12 [4 -29]	12 [4 - 26]	13 [4 - 29]	
**CD4 T cell count (/µl)**				0.67
Median [range]	637 [86 - 1700]	675 [86 - 1700]	613 [204 - 1660]	
**CD4/CD8 ratio**				0.71
Median [range]	0.90 [0.13 - 2.92]	0.88 [0.13 - 2.57]	0.91 [0.15 - 2.92]	
**prior CMV infection**				0.07
yes	7	0	7	
no	101	42	59	
**chronic HBV infection**				1.00
yes	6	2	4	
no	102	40	62	
**chronic HCV infection**				0.89
yes	6	3	3	
no	102	39	63	
*at the time the sample was taken				

Supplementary Table 1: Demographic information of treated PLWH (chronic cohort, on ART for more than 4 years) at the time of sampling, who were fully virologically suppressed. Individuals were grouped into treated PLWH with detectable p24 and those without detectable p24. Statistical significance for continuous variables was assessed using the two-sided Mann-Whitney test for unpaired data, and for categorical variables using the Chi-squared test.

Supplementary Table 2

Treated PLWH ART regimens (n)					
	**NNRTI**	**PI**	**PI/MVC**	**INSTI**	**INSTI/DRV**
p24+	8	10	2	22	0
p24-	21	18	0	23	4

Supplementary Table 2: Distribution of the ART regimens of PLWH at the time of sampling (chronic cohort). Individuals were grouped into treated PLWH with detectable p24 and those without detectable p24.

Supplementary Table 3

	Mean	Male	Female
Age (y)	42 [36 - 56]	-	-
Time on ART to BL (y)	7 [4 -12]	-	-
Time HIV+ to BL (y)*	12 [5 -22]	-	-
Gender (n)	-	11	2

*Data on time from HIV diagnosis to baseline were missing for two patients.

Supplementary Table 3: Demographic information of PLWH who switched from a PI-free ART regimen.

Supplementary Table 4

Activation PBMCs				
**Demographics***	**Overall (n=88)**	p24+ (n=32)	p24- (n=56)	P-value
**Age**				0.73
Median [range]	47 [21 - 74]	47 [26 - 65]	47 [21 - 74]	
**Gender**				1.00
Male	80	29	51	
Female	8	3	5	
**Years on treatment**				0.02
Median [range]	6 [1 -26]	7 [1 - 26]	4 [1 - 21]	
**CD4 T cell count (/µl)**				0.72
Median [range]	631 [86 - 1330]	661 [86 - 1180]	594 [272 - 1330]	
**CD4/CD8 ratio**				0.18
Median [range]	1.04 [0.15 - 2.92]	0.99 [0.15 - 2.57]	1.06 [0.31 - 2.92]	
*at the time the sample was taken				

Supplementary Table 4: Demographic information of treated PLWH (chronic and RV464 cohort) at the time of sampling, who were fully virologically suppressed. Individuals were grouped into treated PLWH with detectable p24 and those without detectable p24. Statistical significance for continuous variables was assessed using the two-sided Mann-Whitney test for unpaired data, and for categorical variables using the Chi-squared test.

Supplementary Table 5

ICS PBMCs				
**Demographics***	**Overall (n=93)**	p24+ (n=34)	p24- (n=59)	P-value
**Age**				0.81
Median [range]	47 [21 -72]	48 [24 - 69]	46 [21 - 72]	
**Gender**				0.92
Male	77	30	47	
Female	6	3	3	
**Years on treatment**				0.09
Median [range]	5 [1 - 26]	7 [1 - 26]	2 [1 - 21]	
**CD4 T cell count (/µl)**				0.97
Median [range]	642 [86 - 1660]	661 [86 - 1180]	614 [249 - 1660]	
**CD4/CD8 ratio**				0.14
Median [range]	1.05 [0.15 - 2.92]	0.93 [0.15 - 2.57]	1.10 [0.31 - 2.92]	
*at the time the sample was taken				

Supplementary Table 5: Demographic information of treated PLWH (chronic and RV464 cohort) at the time of sampling, who were fully virologically suppressed. Individuals were grouped into treated PLWH with detectable p24 and those without detectable p24. Statistical significance for continuous variables was assessed using the two-sided Mann-Whitney test for unpaired data, and for categorical variables using the Chi-squared test.

Supplementary Figure 1

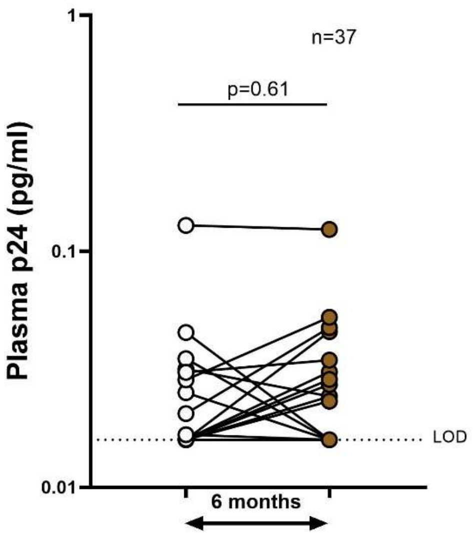

Supplementary Figure 1: Plasma p24 levels were measured in treated PLWH (n =37) at two time points, six months apart. White circles represent p24 measurements at the first time point for each individual, and brown circles represent measurements at the second time point. The dashed line indicates the LOD, set at 16 fg/mL. Within the 37 participants: five individuals (14%) showed increasing p24 levels from below to above the assay's LOD over the 6-month period. four individuals (11%) showed decreasing p24 levels from detectable to below the LOD. The remaining 28 individuals (76%) showed

stable p24 levels, with only minor fluctuations that did not cross the LOD threshold. Statistical difference between time points were assessed using a two-sided sign test. The Y-axis is displayed on a logarithmic scale.

Supplementary Figure 2

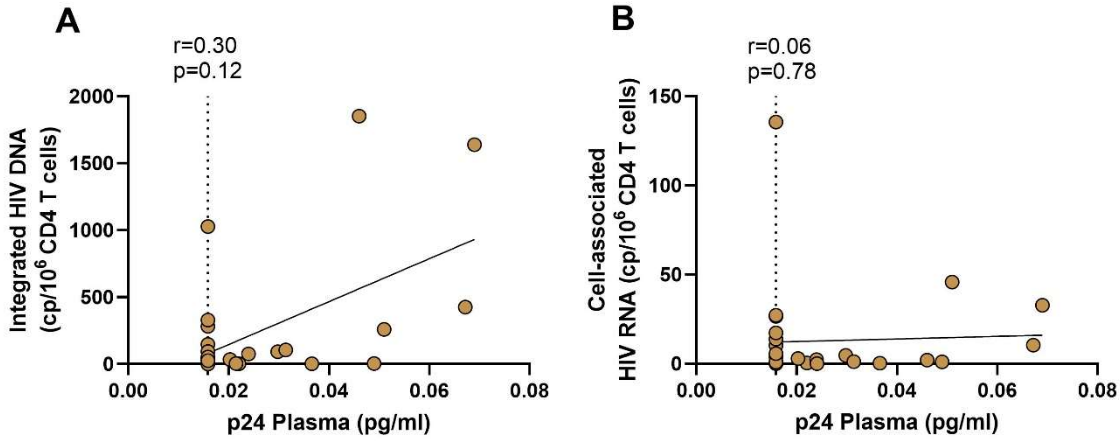

Supplementary Figure 2: Correlation of integrated HIV DNA and cell-associated HIV RNA with plasma p24 levels. **(A)** Plasma p24 levels were correlated with integrated HIV DNA (copies per 10^6 CD4 T cells) in virologically suppressed PLWH (n = 27) from the chronic cohort (n = 24) and the RV464 cohort (n = 3). Among them, 12 individuals had detectable p24 levels and 15 did not. The dashed line indicates the LOD of the p24 assay (16 fg/mL). **(B)** Plasma p24 levels were correlated with cell-associated HIV RNA (copies per 10^6 CD4 T cells) in virologically suppressed PLWH (n = 27) from the chronic cohort (n = 24) and the RV464 cohort (n = 3). Among them, 12 individuals had detectable p24 levels and 15 did not. The dashed line indicates the LOD of the p24 assay (16 fg/mL). Correlations were assessed using two-sided Spearman's rank correlation. Spearman's r > 0 would indicate a positive correlation between the variables.

Supplementary Figure 3

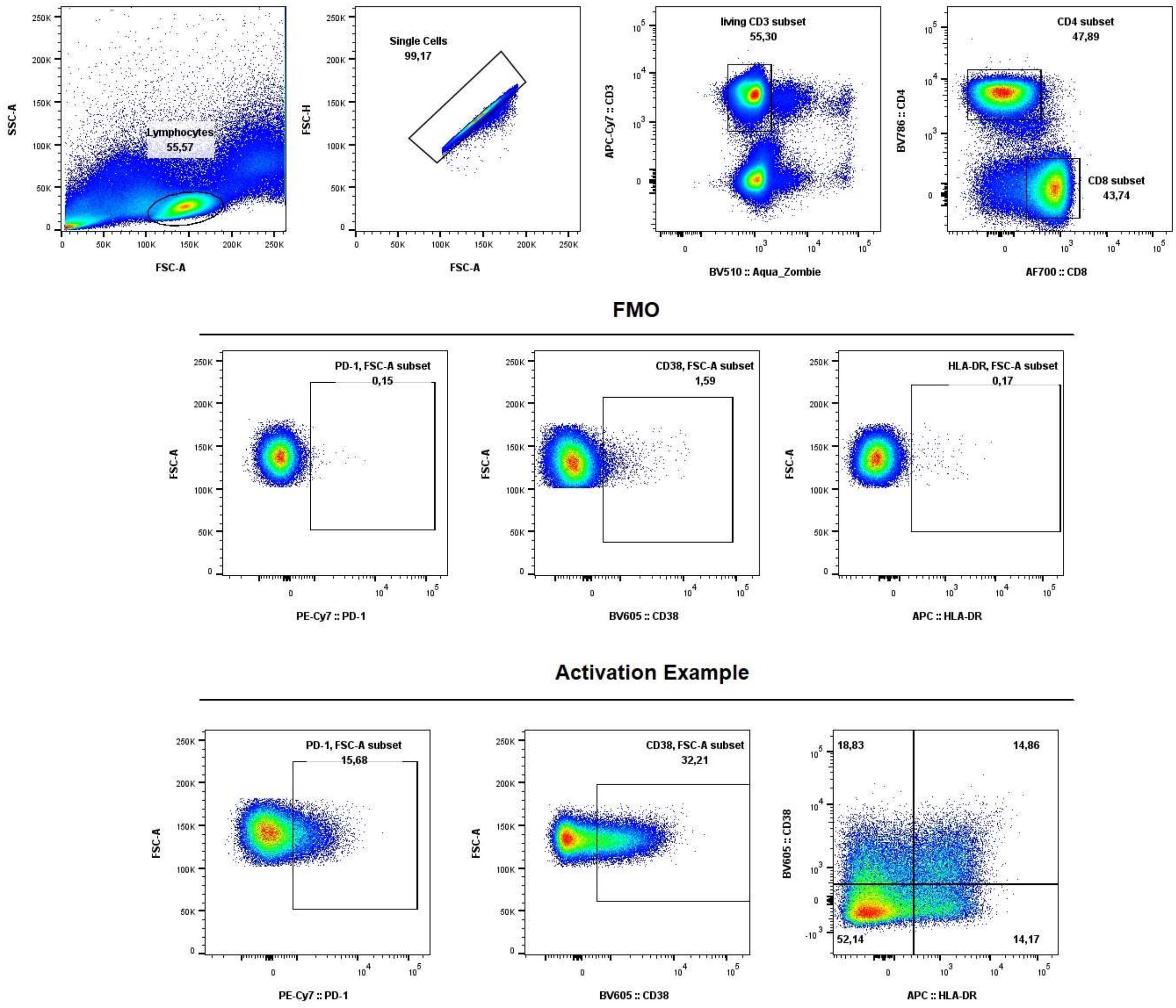

Supplementary Figure 3: Gating strategy for the assessment of T cell activation profile. We first gated on live CD3 T cells, followed by distinguishing between CD4 and CD8 T cells. Next, we gated on CD4 or CD8 T cells expressing PD-1 or CD38, as well as cells co-expressing CD38 and HLA-DR. The frequencies of activation were determined due to fluorescence minus one (FMO) controls.

Supplementary Figure 4

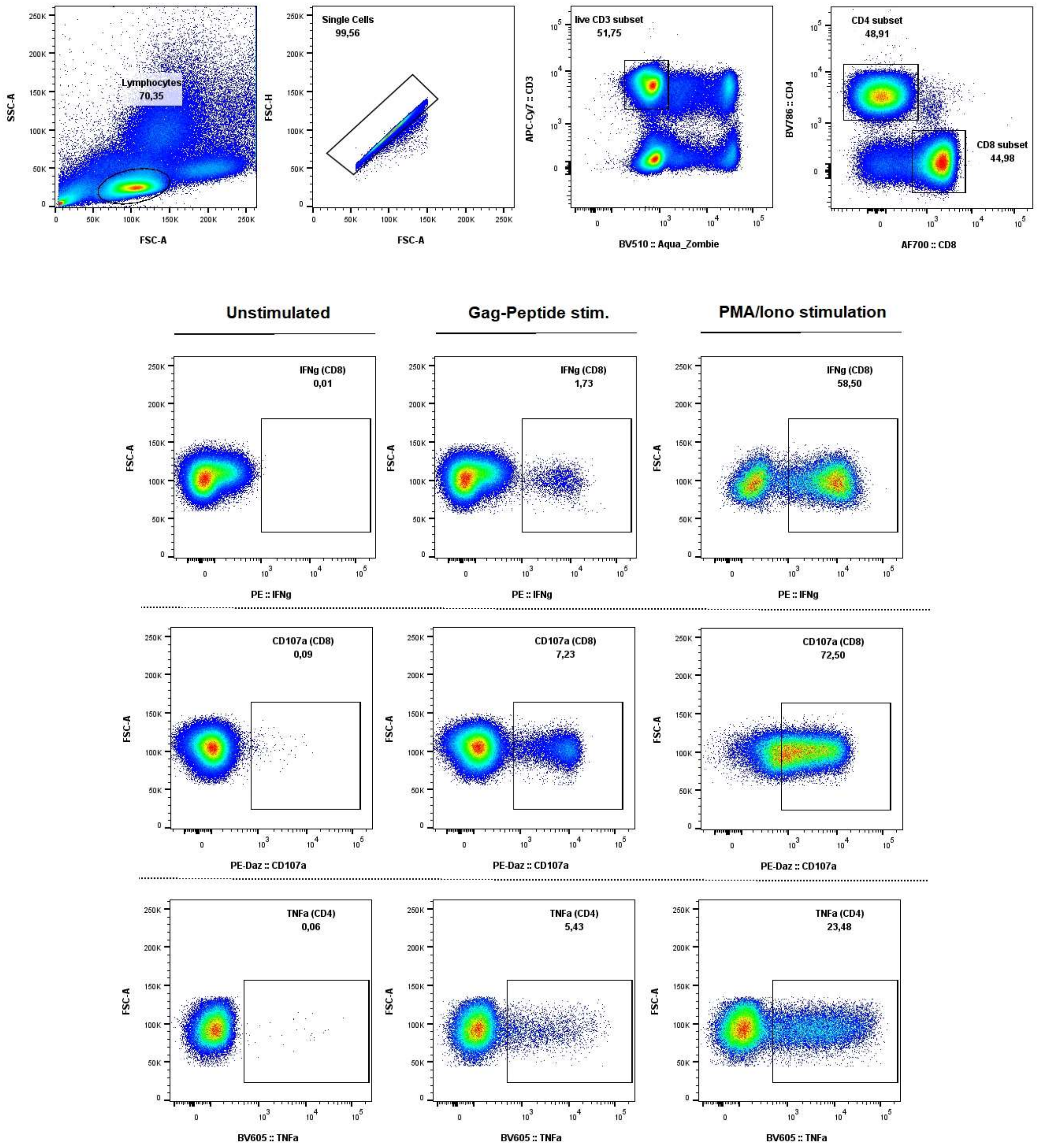

Supplementary Figure 4: Gating strategy for the assessment of T cell cytokine expression profile. We first gated on live CD3 T cells, followed by distinguishing between CD4 and CD8 T cells. Next, we gated on CD4 or CD8 T cells expressing TNF-α, IFN-γ and CD107a according to the negative control. Additionally, the frequencies of antigen-specific T cells were calculated as negative-control-subtracted data. In addition, a positive control for cytokine expression (PBMCs were stimulated with PMA (20 ng/ml) (Sigma-Aldrich) and ionomycin (1 µg/ml)) was added in every run, to ensure a sufficient stimulation capability.
