# Supplementary material for: Ultra-low level HIV p24 drives immune activation in antiretroviral therapy-treated people living with HIV
Source: Commun Med (Lond). 2025 Dec 2;5:510. doi: 10.1038/s43856-025-01261-3 (PMC12672821; doi:10.1038/s43856-025-01261-3)
Supplement: Supplementary file 2 — Description of Additional Supplementary Files [file 43856_2025_1261_MOESM2_ESM.pdf]

Description of Additional Supplementary Files

File name: Supplementary data 1

Description: Cross tabulation for Index Test (Sensitivity and Specificity p24-Assay)

File name: Supplementary data 2

Description: Primer Sequences

File name: Supplementary data 3

Description: Supplementary file presenting the number and percentage of individuals showing increases or decreases in p24 levels, values above or below the LOD, or no changes at all.

File name: Supplementary data 4-8

Description: Source data behind the graphs in the paper
